# Scuba: scalable kernel-based gene prioritization

**DOI:** 10.1186/s12859-018-2025-5

**Published:** 2018-01-25

**Authors:** Guido Zampieri, Dinh Van Tran, Michele Donini, Nicolò Navarin, Fabio Aiolli, Alessandro Sperduti, Giorgio Valle

**Affiliations:** 1 CRIBI Biotechnology Center, University of Padova, viale G. Colombo, 3, Padova, Italy; 2 Department of Women’s and Children’s Health, University of Padova, via Giustiniani, 3, Padova, Italy; 3 Department of Mathematics, University of Padova, via Trieste, 63, Padova, Italy; 4 Istituto Italiano di Tecnologia, Via Morego, 30, Genoa, Italy; 5 Department of Biology, University of Padova, viale G. Colombo, 3, Padova, Italy

**Keywords:** Gene prioritization, Genetic disease, Kernel methods, Semi-supervised learning

## Abstract

**Background:**

The uncovering of genes linked to human diseases is a pressing challenge in molecular biology and precision medicine. This task is often hindered by the large number of candidate genes and by the heterogeneity of the available information. Computational methods for the prioritization of candidate genes can help to cope with these problems. In particular, kernel-based methods are a powerful resource for the integration of heterogeneous biological knowledge, however, their practical implementation is often precluded by their limited scalability.

**Results:**

We propose Scuba, a scalable kernel-based method for gene prioritization. It implements a novel multiple kernel learning approach, based on a semi-supervised perspective and on the optimization of the margin distribution. Scuba is optimized to cope with strongly unbalanced settings where known disease genes are few and large scale predictions are required. Importantly, it is able to efficiently deal both with a large amount of candidate genes and with an arbitrary number of data sources. As a direct consequence of scalability, Scuba integrates also a new efficient strategy to select optimal kernel parameters for each data source. We performed cross-validation experiments and simulated a realistic usage setting, showing that Scuba outperforms a wide range of state-of-the-art methods.

**Conclusions:**

Scuba achieves state-of-the-art performance and has enhanced scalability compared to existing kernel-based approaches for genomic data. This method can be useful to prioritize candidate genes, particularly when their number is large or when input data is highly heterogeneous. The code is freely available at https://github.com/gzampieri/Scuba.

**Electronic supplementary material:**

The online version of this article (10.1186/s12859-018-2025-5) contains supplementary material, which is available to authorized users.

## Background

The identification of the genes underlying human diseases is a major goal in current molecular genetics research. Dramatic progresses have been made since the 1980s, when only a few DNA loci were known to be related to disease phenotypes. Nowadays opportunities for the diagnosis and the design of new therapies are progressively growing, thanks to several technological advances and the application of statistical or mathematical techniques. For instance, positional cloning has allowed to map a vast portion of known Mendelian diseases to their causative genes [1, 2]. However, despite the huge advances, much remains to be discovered. On December 21st 2016, the Online Mendelian Inheritance in Man database (OMIM) registered 4908 Mendelian phenotypes of known molecular basis and 1483 Mendelian phenotypes of unknown molecular origin [3]. Moreover, 1677 more phenotypes were suspected to be Mendelian. But it is among oligogenic and poligenic (and multifactorial) pathologies that the most remains to be elucidated: for the majority of them, only a few genetic loci are known [1, 2].

Independently of the type of disease, the search of causative genes usually concerns a large number of suspects. It is therefore necessary to recognise the most promising candidates to submit to additional investigations, as experimental procedures are often expensive and time consuming. Gene prioritization is the task of ordering genes from the most promising to the least. In traditional genotype-phenotype mapping approaches - as well as in genome-wide association studies - the first step is the identification of the genomic region(s) wherein the genes of interest lie. Once the candidate region is identified, the genes there residing are prioritized and finally analysed for the presence of possible causative mutations [1]. More recently, in new generation sequencing studies this process is inverted as the first step is the identification of mutations, followed by prioritization and final validation [4]. Prioritization criteria are usually based on functional relationships, co-expression and other clues linking genes together. In general, all of them follow the “guilt-by-association” principle, i.e. disease genes are sought by looking for similarities to genes already associated to the pathology of interest [1].

In the last few years, computational techniques have been developed to aid researchers in this task, applying both statistics and machine learning [5]. Thanks to the advent of high-throughput technologies and new generation sequencing, a huge amount of data is in fact available for this kind of investigations. In particular, computational methods are essential for multi-*omics* data integration, that has been recognised as a valuable strategy for understanding genotype-phenotype relationships [6]. In fact, clues are often embedded in different data sources and only their combination leads to the emergence of informative patterns. Furthermore, incompleteness and noise of the single sources can be overcome by inference across multiple levels of knowledge.

Several popular algorithms for pattern analysis are based on *kernels*, which are mathematical transformations that permit to estimate the similarity among items (in our case genes) taking into account complex data relations [7]. Importantly, kernels provide a universal encoding for any kind of knowledge representation, e.g. vectors, trees or graphs. When data integration is required, a multiple kernel learning (MKL) strategy allows a data-driven weighting/selection of meaningful information [8]. The goal of MKL is indeed to learn optimal kernel combinations starting from a set of predefined kernels obtained by various data sources. Through MKL the issue of combining different data types is then solved by converting each dataset in a kernel matrix.

Numerous MKL approaches have been proposed for the integration of genomic data [9, 10] and some of them have been applied to gene prioritization [11–14]. De Bie et al. formulated the problem as a one-class support vector machine (SVM) optimization task [11], while Mordelet and Vert tackled it through a biased SVM in a *positive-unlabelled* framework [13, 15]. Recently, Zakeri et al. proposed an approach for learning non-linear log-euclidean kernel combinations, showing that it can more effectively detect complementary biological information compared to linear combinations-based approaches [14]. However, as highlighted in a recent work by Wang et al. [9], current methods share two limitations: high computational costs - given by a (at least) quadratic complexity in the number of training examples - and the difficulty to predefine optimal kernel functions to be fed to the MKL machine.

In this work we tackle these issues by proposing a novel scalable gene prioritization method based on a particular MKL approach [16]. By this approach, the optimal kernel is efficiently computed by maximizing the distance between positive and negative examples and optimizing the margin distribution [17]. This permits to obtain a high scalability relatively to the number of kernels, with a linear time complexity and a practically constant memory requirement. However, this approach assumes comparable label noise in the two example distributions, which does not reflect the case in consideration. Moreover, it does not scale with the number of training examples. Here we introduce a new algorithm, specifically adapted to a *positive-unlabelled* unbalanced framework and we apply it to gene prioritization for the first time. The new learning algorithm has an additional gain in scalability that comes particularly useful when large numbers of genes have to be prioritized. This scalability allows us to transform each data source by multiple kernels and alleviates the issue of defining appropriate base kernels for each source. We called the proposed method Scuba (SCalable UnBAlanced gene prioritization).

From an experimental point of view, here we focus on the integration of multiple gene networks whose edges symbolize functional relationships from heterogeneous sources and we employ two different test settings. In the first setting, we reproduce the procedure presented in a previous work by Chen et al. [18], built upon cross-validation experiments [19] on collections of known disease genes. This kind of evaluation is useful to compare different methods, but results may suffer from overestimation due to the reliance of many data repositories on medical literature or external data sources like OMIM [3]. Such dependence introduces a bias that may favour the retrieval of known disease genes. Thus, as a second validation we employ a more realistic setting, following a previous evaluation of gene prioritization tools by Börnigen et al. [20]. Here performance measures focus on the ability of predicting disease genes discovered subsequently to the last update of datasets.

Overall, we compare Scuba with other 14 gene prioritization systems, including other 2 kernel-based methods and 8 web tools. We find that Scuba has competitive accuracy and in particular yields the best results in genome-wide prioritizations, showing its value for large-extent applications.

## Methods

In this section, we first introduce and formalize some concepts that will be used throughout this paper. Then, we present the proposed approach in detail.

**Disease gene prioritization**: Let us consider a set of genes $\mathcal {G} = \lbrace g_{1}, g_{2}, \ldots, g_{N} \rbrace $ that represents either the global set of genes in the genome or a subset of it. Given another set $\mathcal {P} = \lbrace g_{1}, g_{2}, \ldots, g_{m} \rbrace, \, \mathcal {P} \subset \mathcal {G}$ containing genes known to be associated to a genetic disease, gene prioritization is the task that aims to rank genes in the set of candidates $\mathcal {U} = \mathcal {G}\! \!\setminus \!\! \mathcal {P}= \lbrace g_{m+1}, g_{m+2}, \ldots, g_{N} \rbrace $ according to their likelihood of being related to that disease. Genes in $\mathcal {P}$ are labelled as *positive* and represent a secure source of information. In contrast, candidate genes in $\mathcal {U}$ are technically *unlabelled*, as we expect that some of them may be associated to the disease but we do not know which ones. Under this notation, this problem can be posed as a *positive-unlabelled* (PU) learning task [13, 15].

**Kernel**: *Kernels* can be informally seen as similarity measures between pairs of data examples. Mathematically, such similarities are defined by inner products between vectors of corresponding examples in a Hilbert space $\mathcal {H}$, without the need of an explicit transformation to that space. A kernel function *k* on $\mathcal {X} \times \mathcal {X} $ is then formally defined as:

$$\begin{array}{*{20}l} k: \mathcal{X} &\times \mathcal{X} \longrightarrow \mathcal{R}\\ k(x_{1}, x_{2}) &= \mathcal{<}\phi(x_{1}),\phi(x_{2})\mathcal{>} \,, \end{array} $$

where $x_{1}, x_{2} \in \mathcal {X}$, *ϕ* is a mapping $\phi :\mathcal {X} \longrightarrow \mathcal {H}$ and *k* needs to be (1) symmetric, i.e. *k*(*x*_1_,*x*_2_)=*k*(*x*_2_,*x*_1_) (2) semi-definite, i.e. the kernel matrix defined by *k*_*ij*_=*k*(*x*_*i*_,*x*_*j*_) has all eigenvalues ≥ 0. Kernels can be used to define similarities starting from various data types, like graph nodes.

**Graph node kernel**: A graph *G*=(*V*,*E*) is a structure consisting of a node set *V*={*v*_1_,…,*v*_*N*_} and an edge set *E*={(*v*_*i*_,*v*_*j*_)|*v*_*i*_,*v*_*j*_∈*V*)}. A graph node kernel aims at defining a similarity between any couples of nodes in a graph. A considerable number of graph node kernels have been introduced. The most popular is the diffusion kernel [21] which is based on the heat diffusion phenomenon. The key idea is to allow a given amount of *heat* on each node and let it *diffuse* through the edges. The similarity between two nodes *v*_*i*_,*v*_*j*_ is then measured as the amount of heat starting from *v*_*i*_ and reaching *v*_*j*_ over an infinite time interval. In the diffusion kernel the heat flow is proportional to the number of paths connecting two nodes, introducing a bias that penalizes peripheral nodes with respect to central ones. This problem is tackled by a modified version called Markov exponential diffusion kernel (MEDK) [22] where a Markov matrix replaces the adjacency matrix. Another kernel called Markov diffusion kernel (MDK) [23], exploits the notion of *diffusion distance*, a measure of similarity between patterns of heat diffusion. The regularized Laplacian kernel (RLK) [24] implements instead a normalized version of the random walk with restart model and defines the node similarity as the number of paths connecting two nodes with different lengths.

### Scalable multiple kernel learning: EasyMKL

We approach the problem of disease gene prioritization by employing a graph-based integration in which we use graph node kernels to extract gene information and encode it in the form of kernel matrices. However, a big challenge is how to effectively combine kernels when building predictive systems. This challenge can be solved by MKL. In the following, we first formalize the MKL problem and we then briefly introduce a scalable MKL algorithm named EasyMKL [16].

Given a set of pre-defined kernels, multiple kernel learning is a task that aims at finding an optimal kernel combination:

1 $$ \textbf{K} = \psi(\textbf{K}_{1}, \textbf{K}_{2},\ldots, \textbf{K}_{R}) \,.  $$

Recently, many MKL methods have been proposed [8, 9]. However, most of them require a long computation time and a high memory consumption, especially when the number of pre-defined kernels is high. To tackle these limitations, a scalable multiple kernel learning named EasyMKL has been previously proposed [16]. This method focuses on learning a linear combination of the input kernels with positive linear coefficients, namely

2 $$ \textbf{K} = \sum_{r=1}^{R} {\eta_{r} \textbf{K}_{r}}, \ \eta_{r} \geq 0 \,,  $$

where *η*=(*η*_1_,…,*η*_*R*_) is the coefficient vector. In a fully supervised binary task, EasyMKL computes the optimal kernel by maximizing the distance between positive and negative examples. The base learner is a kernel-based approach for the optimization of the margin distribution in binary classification or ranking [17].

In order to present its formulation, let us first define the probability distribution $\gamma \in \mathbb {R}^{N}_{+}$ representing weights assigned to training examples and living in the domain $\Gamma = \left \{ \gamma \in \mathbb {R}^{N}_{+} | \sum _{i \in \mathcal {P}} \gamma _{i}=1, \sum _{i \in \mathcal {N}} \gamma _{i}=1\right \}$, where $\mathcal {N}$ is the set of negative examples. From this definition, it follows that any element *γ*∈*Γ* represents a pair of points in the input space: the first one is constrained to the convex hull of positive training examples and the second one to the convex hull of negative training examples. As stated above, EasyMKL maximizes the distance between positive and negative examples, optimizing the margin distribution at the same time. Under this notation, the task can be posed as a min-max problem over variables *γ* and *η* as follows:

3 $$ \underset{\eta:\| \eta \|_{2} \leq 1}{\text{max}}\underset{\gamma \in \Gamma}{\text{min}}\, (1 - \lambda) \gamma^{\top} \textbf{Y} \left(\sum_{r}{\eta_{r} \textbf{K}_{r}}\right)\textbf{Y} \gamma + \lambda \, \gamma^{\top} \gamma \,.  $$

Here **Y** is a diagonal matrix containing the vector of example labels, +1 for the positive and -1 for the negative. Optimization of the first term alone leads to an optimal probability distribution *γ*^∗^ representing the two nearest points in the convex hulls of positive and negative examples, equally to a hard SVM task using a kernel **K** [17]. The second term represents a quadratic regularization over *γ* whose objective solution is the squared distance between positive and negative centroids in the feature space. The regularization parameter *λ*∈[0,1] permits to tune the objective to optimize, by balancing between the two critical values *λ*=0 and *λ*=1. When *λ*=0 we obtain a pure hard SVM objective, while when *λ*=1 we get a centroid-based solution.

It can be shown that this problem has analytical solution in the *η* variable, so that the previous expression can be reshaped into:

4 $$ \underset{\gamma \in \Gamma}{\text{min}} \, (1 - \lambda) \gamma^{\top} \textbf{Y} \textbf{K}^{s} \textbf{Y} \gamma + \lambda \, \gamma^{\top} \gamma \,,  $$

where $\textbf {K}^{s}=\sum _{r}^{R}\textbf {K}_{r}$ is the sum of the pre-defined kernels. This minimization can be efficiently solved and only requires the sum of the kernels. The computation of the kernel summation can be easily implemented incrementally and only two matrices need to be stored in memory at a time. As shown in [16], EasyMKL can deal with an arbitrary number of kernels using a fixed amount of memory and a linearly increasing computation time.

Once the problem in Eq. 4 is solved, we have an optimal distribution *γ*^∗^ and we are able to obtain the optimal kernel weights $\eta _{r}^{*}$ by using the formula:

5 $$ \eta_{r}^{*} = \frac{\gamma^{*} \textbf{Y} \textbf{K}_{r} \textbf{Y} \gamma^{*}}{\sum_{r=1}^{R} \gamma^{*} \textbf{Y} \textbf{K}_{r} \textbf{Y} \gamma^{*}} \,.  $$

The optimal kernel is thus evaluated as $ \textbf {K}^{*} = \sum _{r}^{R} \eta _{r}^{*} \textbf {K}_{r}$. Finally, by replacing **K**^*s*^ with **K**^∗^ in Eq. 4, we can get the final probability distribution *γ*^∗^.

### Unbalanced multiple kernel learning: Scuba

In the previous section we introduced EasyMKL, a scalable, efficient kernel integration approach. However, the gene prioritization task has two additional issues that complicate the work. First, our learning setting is not fully supervised: an assumption is that there are some positive examples hidden among the negatives and we want to retrieve them. Thus, we have the certainty about positive examples but not about negative ones. Second, the number of known disease genes is typically much smaller than the number of candidates, making the problem strongly unbalanced. For these reasons, inspired by a previous work [25] we propose a new MKL algorithm based on EasyMKL that not only inherits its scalability, but also efficiently deals with an unbalanced setting.

In order to clearly present our method, we first need to highlight the different contributions given by positive and unlabelled examples. Therefore, we define **K**^+^,**K**^−^ and **K**^+−^ the sub-matrices of **K**^*s*^ pertaining to positive-positive, unlabelled-unlabelled and positive-unlabelled example pairs, respectively. Schematically, we have:

$$\textbf{K}^{s} = \left(\begin{array}{cc} \textbf{K}^{+} & \textbf{K}^{+-}\\ \textbf{K}^{-+} & \textbf{K}^{-}\\ \end{array} \right) \,. $$

being **K**^−+^ the transpose of **K**^+−^. In other words, **K**^+^ contains similarities among positive examples $g_{i} \in \mathcal {P}, i = 1, \ldots m, \textbf {K}^{-}$ contains similarities among unlabelled examples $g_{j} \in \mathcal {U}, j = m+1, \ldots N$ and **K**^+−^ includes similarities between positive-unlabelled example pairs. In the same way, we define *γ*_+_ and *γ*_−_ as the probability vectors associated to positive and unlabelled examples, respectively.

Under this change of variables, we reformulate the problem as:

$$\begin{aligned} \underset{\gamma \in \Gamma}{\text{min}} \, \gamma_{+}^{\top} \textbf{K}^{+} \gamma_{+} - 2 \,\gamma_{+}^{\top} \textbf{K}^{+-} \gamma_{-} + \gamma_{-}^{\top} \textbf{K}^{-} \gamma_{-} \\+ \lambda_{+} \gamma_{+}^{\top} \gamma_{+} + \lambda_{-} \gamma_{-}^{\top} \gamma_{-} \,. \end{aligned} $$

In this new formulation, the original EasyMKL problem is obtained by setting $\lambda _{+} = \lambda _{-} = \frac {\lambda }{1-\lambda }$. However, due to the unbalanced PU nature of the problem, we are interested in using two different regularizations among positive and unlabelled examples. In our case, we decide to fix a priori the regularization parameter *λ*_−_=+*∞*, corresponding to fixing *λ*=1 over unlabelled examples only. Then, the solution of part of the objective function is defined by the uniform distribution $\gamma _{-} = \left (\frac {1}{n},\frac {1}{n},\ldots \frac {1}{n}\right) \equiv u$, where *n*=*N*−*m* is the number of unlabelled examples.

We inject this analytic solution of part of the problem in our objective function as

$$\begin{aligned} \underset{\gamma \in \Gamma^{+}}{\text{min}} \, \gamma_{+}^{\top} \textbf{K}^{+} \gamma_{+} - 2 \,\gamma_{+}^{\top} \textbf{K}^{+-} u + u^{\top} \textbf{K}^{-} u \\+ \lambda_{+} \gamma_{+}^{\top} \gamma_{+} + \lambda_{-} u^{\top}u \,, \end{aligned} $$

where $\Gamma ^{+}\! =\! \left \{ \gamma \in \mathcal {R}_{+}^{m} | \sum _{i = 1}^{m} \gamma _{i} = 1, \gamma _{j} = 1/n \ \forall \, j = m+1,\right. \left. \ldots N \right \}$ is the probability distribution domain where the distributions over the unlabelled examples correspond to the uniform distribution. It is trivial that *u*^⊤^**K**^−^*u* and *λ*_−_*u*^⊤^*u* are independent from the *γ*_+_ variable. Then, they can be removed from the objective function obtaining

6 $$ \underset{\gamma \in \Gamma^{+}}{\text{min}} \, \gamma_{+}^{\top} \textbf{K}^{+} \gamma_{+} - 2 \, \gamma_{+}^{\top} \textbf{K}^{+-} u + \lambda_{+} \gamma_{+}^{\top} \gamma_{+} \,.  $$

In this expression, we only need to consider the entries of the kernel **K**^*s*^ concerning the positive set, avoiding all the entries with indices in the unlabelled set. The complexity becomes quadratic in the number of positive examples *m*, which is always much smaller than the number of examples to prioritize. Moreover, this algorithm still depends linearly on the number of kernels *R* and the overall time complexity is then $\mathcal {O}(m^{2} \cdot R)$. In this way, we greatly simplify the optimization problem, while being able to take into account the diverse amount of noise present in positive and unlabelled example sets.

Like in the previous section, after solving the problem of Eq. 6 we use Eq. 5 to compute the optimal kernel weights $\eta _{r}^{*}$. Next, we solve again the Scuba optimization problem to get the final optimal probability distribution *γ*^∗^. Test genes are evaluated by taking the weighted sum over all rectangular test kernel matrices $\mathbf {K}_{r}^{t}$, where rows and columns represent test and training genes respectively. In formula:

$$ \mathbf{K}^{t*} = \sum_{r=1}^{R} \eta_{r}^{*} \mathbf{K}_{r}^{t} \,, $$

The likelihood of association to the disease for any test gene *g*_*i*_ is given by the score *s*_*i*_ defined as

7 $$ s_{i} = \sum_{j} y_{j} \gamma_{j}^{*} \textbf{K}_{ij}^{t*} \,,  $$

where *y*_*j*_ and $\gamma _{j}^{*}$ are the label and optimal weight of any training example *g*_*j*_ and $\textbf {K}_{ij}^{t*}$ is the optimal kernel value between *g*_*j*_ and the test gene *g*_*i*_. In other words, *s*_*i*_ is the weighted sum over the similarities between the test gene *g*_*i*_ and all genes in the training set. Once we get the scores for test genes, they can be prioritized based on their score values.

### Base kernels selection

We leverage the scalability achieved by the new algorithm to ease the optimization of base kernels. As a general practical case, we start from a set of data sources $\mathcal {S} = \lbrace S_{1}, S_{2},\ldots, S_{L} \rbrace $ representing various levels of biological information. We first construct a set of corresponding graphs derived from the set of data sources *S* to obtain a set of graphs $\mathcal {T} = \lbrace G_{1}, G_{2}, \ldots, G_{L}\rbrace $. We then apply different kernels with different parameter values on each $G_{i} \in \mathcal {T}$. As a consequence, for each graph *G*_*i*_, we get a set of kernel matrices $\mathcal {K}_{i} = \lbrace \textbf {K}_{i1}, \textbf {K}_{i2},\ldots, \textbf {K}_{iH} \rbrace $. By collecting all kernels from all $\mathcal {K}_{i}$, we achieve a final kernel matrix set $\mathcal {K}$ comprising *L*·*H* matrices. Next, all matrices in $\mathcal {K}$ and gene sets $\mathcal {P}$ and $\mathcal {U}$ are fed into Scuba to obtain the optimal kernel **K**^∗^. In this way, we directly use MKL to perform an automatic selection of optimal kernel parameters. The final kernel and the disease gene set $\mathcal {P}$ are then employed to train a model, which is used to generate a score list for candidate genes in $\mathcal {U}$ through Eq. 7. The score assigned to a candidate expresses the likelihood of it being associated to the disease.

### Experimental workflow

We employed Scuba to prioritize candidate genes starting from multiple gene networks, obtained by various data sources. We transformed every network by means of multiple graph node kernels as explained in the previous section. In the cross-validation experimental setting we used MEDK to estimate the similarity among genes, just like in [18]. In the unbiased setting we used MDK and RLK, selected by validating on training sets.

In both settings, we fixed the number of kernel matrices per data source *H*=3 and learned the regularization parameter *λ*_+_ by employing k-fold cross validation on the training set, using the the grid of values {0, 0.1, 0.2, …,1}. Kernel parameter values were set as follows: {0.01, 0.04, 0.07} for MEDK, as suggested in [26] and used in [22], {2, 4, 6} for MDK and {1, 10, 100} for RLK, as suggested in [23].

### Data sources

We employed several biological data sources to test Scuba, presented in the following.

- **Human protein reference database** (**HPRD**) [27]. The HPRD resource provides protein interaction data which we implement as an unweighted graph, where genes are linked if their corresponding proteins interact.
- **BioGPS** [28]. It contains expression profiles for 79 human tissues, which are measured by using the Affymetrix U133A array. Gene co-expression, defined by pairwise Pearson correlation coefficients (PCC), is used to build an unweighted graph. A pair of genes are linked by an edge if the PCC value is larger than 0.5.
- **Pathways**. Pathway datasets are obtained from the database of KEGG [29], Reactome [30], PharmGKB [31] and PID [32], which contain 280, 1469, 99 and 2679 pathways, respectively. A pathway co-participation network is constructed by connecting genes that co-participate in any pathway.
- **String** [33]. The String database gathers protein information covering seven levels of evidence: genomic proximity in procaryotes, fused genes, co-occurrence in organisms, co-expression, experimentally validated physical interactions, external databases and text mining. Overall, these aspects focus on functional relationships that can be seen as edges of a weighted graph, where the weight is given by the reliability of that relationship. To perform the unbiased evaluation we employed the version 8.2 of String, from which we extracted functional links among 17,078 human genes.

The first three datasets were obtained directly from Chen et al. [18], already preprocessed in such a way that all of them represent exactly the same 7311 genes. We employed this data without any further processing.

Known gene-disease associations employed in the cross-validation experimental setting were taken from a work of Goh et al. which defines classes of related diseases [34]. Training and candidate gene sets used in the second set of experiments (“Unbiased evaluation” section) were obtained from the supplementary material of the unbiased evaluation of gene prioritization tools performed by Börnigen et al. [20]. Finally, gene-disease associations from the Human Phenotype Ontology were used, belonging to builds 29 and 117 [35].

### Other kernel-based gene prioritization methods

We compare Scuba with other two kernel methods for gene prioritization. The first one implements a one class approach to MKL, slightly modifying the formulation of the method of De Bie et al. [11]. In the corresponding work [12], authors show that this newer approach reaches higher performances in ranking. In the following, we refer to it as MKL1class. The second method we consider is ProDiGe, a PU approach that combines MKL and multitask learning [13]. We focus on its first version without multitask learning, as our purpose is to study performances in terms of the MKL framework. We ran ProDiGe using the default parameters indicated in the corresponding paper: number of bagging iterations *B*=30 and regularization parameter *C*=1. In the same way, we set the regularization parameter *ν*=0.5 for MKL1class.

## Results

In this section, we describe the tests made to evaluate our proposed method, which follow two different experimental procedures. In the first setting, we aim at estimating Scuba performance in a standard validation framework. In the second setting we evaluate it by an unbiased approach, making a comparison with prioritization tools available on the web and with two state-of-the-art kernel-based methods.

### Cross-validation

As a first evaluation of Scuba, we followed the experimental protocol used by Chen et al. to test predictive performance of other prioritization methods [18]. In this setting, we employed three data sets: BioGPS, HPRD and Pathways, which we borrowed from the authors of the work. To perform the experiments, we employed known gene-disease associations from OMIM, grouped into 20 classes on the basis of disease relatedness by Goh et al. [34]. Among those classes we selected the 12 with at least 30 confirmed genes. We then built a training set consisting of a positive set $\mathcal {P}$ and an unlabelled set $\mathcal {U}$ for each of them. $\mathcal {P}$ contains all its disease gene members. $\mathcal {U}$ is constructed by randomly picking genes from known disease genes such that $\vert \mathcal {U} \vert = \frac {1}{2} \vert \mathcal {P} \vert $. The unlabelled genes relate to at least one disease class, but do not relate to the current class. We chose the genes in $\mathcal {U}$ from the other disease genes because we assumed that they were less likely to be associated to the considered class. In fact, disease genes are generally more studied and a potential association has more chances to have already been identified.

After that, leave-one-out cross validation was used to evaluate the performance of the algorithm. Iteratively, every gene in the training set was selected to be the test gene and the remaining genes in $\mathcal {P}$ and $\mathcal {U}$ were used to train the model. Once the model was trained, a score list for the test gene and all genes associated to no disease was computed. Then, we computed a decision score for each test gene representing the percentage of candidate genes ranked lower than it. We collected all decision scores for every gene in all disease classes to form a global decision score list. The performance of Scuba was measured by calculating the area under the curve (AUC) in the receiver-operating-characteristic plot obtained from the decision score list. The AUC expresses the probability that a randomly chosen disease gene is ranked above a randomly picked non-disease gene for any disease class.

Table 1 illustrates the performance of different techniques in this experimental setting reported by Chen et al. [18], and the performance of our proposed method. In the second column we show the significance of the difference between reported AUCs and Scuba AUC, assessed by means of separate pairwise comparisons (i.e. we control the comparison-wise error rate), according to the statistical test proposed by Hanley and McNeil in [36]. Scuba performs significantly better than the other methods, getting an AUC around 3.6% greater than the second best performing technique, F3PC.

**Table 1:** The performance of different techniques in the experimental setting of Chen et al. [18] expressed in terms of AUC

Method	AUC	*p*-value
Scuba	0.876	-
F3PC [18]	0.830	1.39 ·10^−4^ *
MRF [22]	0.731	< 10^−6^ *
DIR [26]	0.716	< 10^−6^ *
GeneWanderer [43]	0.711	< 10^−6^ *

Except for our proposed method Scuba, these results were taken from that work. The *p*-values indicate significance of the pairwise AUC differences with respect to Scuba AUC [36]. Asterisks indicate significance of the test (*p*-value < 0.05)

### Unbiased evaluation

Although the previous evaluation is useful to compare Scuba with other methods, predictive performance in cross-validation experiments may be inflated compared to real applications. Indeed, the retrieval of known disease genes can be facilitated by various means. One mean is the crosstalk between data repositories: for example, KEGG [29] draws its information also from medical literature. Moreover, often the discovery of the link between a gene and a disease coincides with the discovery of a functional annotation or of a molecular interaction. In practice, instead, researchers are interested in novel associations, which in most cases are harder to find due to a lack of information around them.

In order to achieve a thorough evaluation of Scuba, we tested it in a more realistic setting, following the work of Börnigen et al. [20]. In this study, eight gene prioritization web tools were benchmarked as follows. Newly discovered gene-disease associations were collected over a timespan of six months, gathering 42 test genes associated to a range of disorders. As soon as a new association was discovered, each web tool was queried with a disorder-specific set of positive genes $\mathcal {P}$ to prioritize a set of candidates $\mathcal {U}$ containing the corresponding test gene (or to prioritize the whole genome where possible). In other words, the test gene was treated as unlabelled to simulate the re-discovery of its association with the disease. Rank positions of the 42 test genes were ultimately used to assess the ability of the tools to successfully prioritize disease genes. The idea behind this procedure is to anticipate the integration of the associations in the data sources and so avoid biased predictions.

In order to test Scuba in this setting, we backdated our data to a time prior to May 15, 2010 by employing String v8.2 data [33]. After that, we recovered positive sets and test genes from the original publication and we followed its experimental protocol as follows [20]. We performed prioritizations for each test gene in two distinct cases: genome-wide and candidate set-based prioritizations. In any genome-wide prioritization all genes in the String dataset - except those in $\mathcal {P}$ - belong to $\mathcal {U}$ and were prioritized. In any candidate set-based prioritization, the set of candidates $\mathcal {U}$ was constructed by considering all genes with Ensembl [37] gene identifier within the chromosomal regions around the test gene, in order to get on average 100 candidates. In both cases, we normalized the ranking positions over the total number of considered genes in order to get median, mean and standard deviation of the normalized ranks for test genes. We also computed the true positive rate (TPR) relatively to some representative thresholds (5%, 10% and 30% of the ranking) and the AUC obtained by averaging over the 42 prioritizations.

**Fig. 1:**
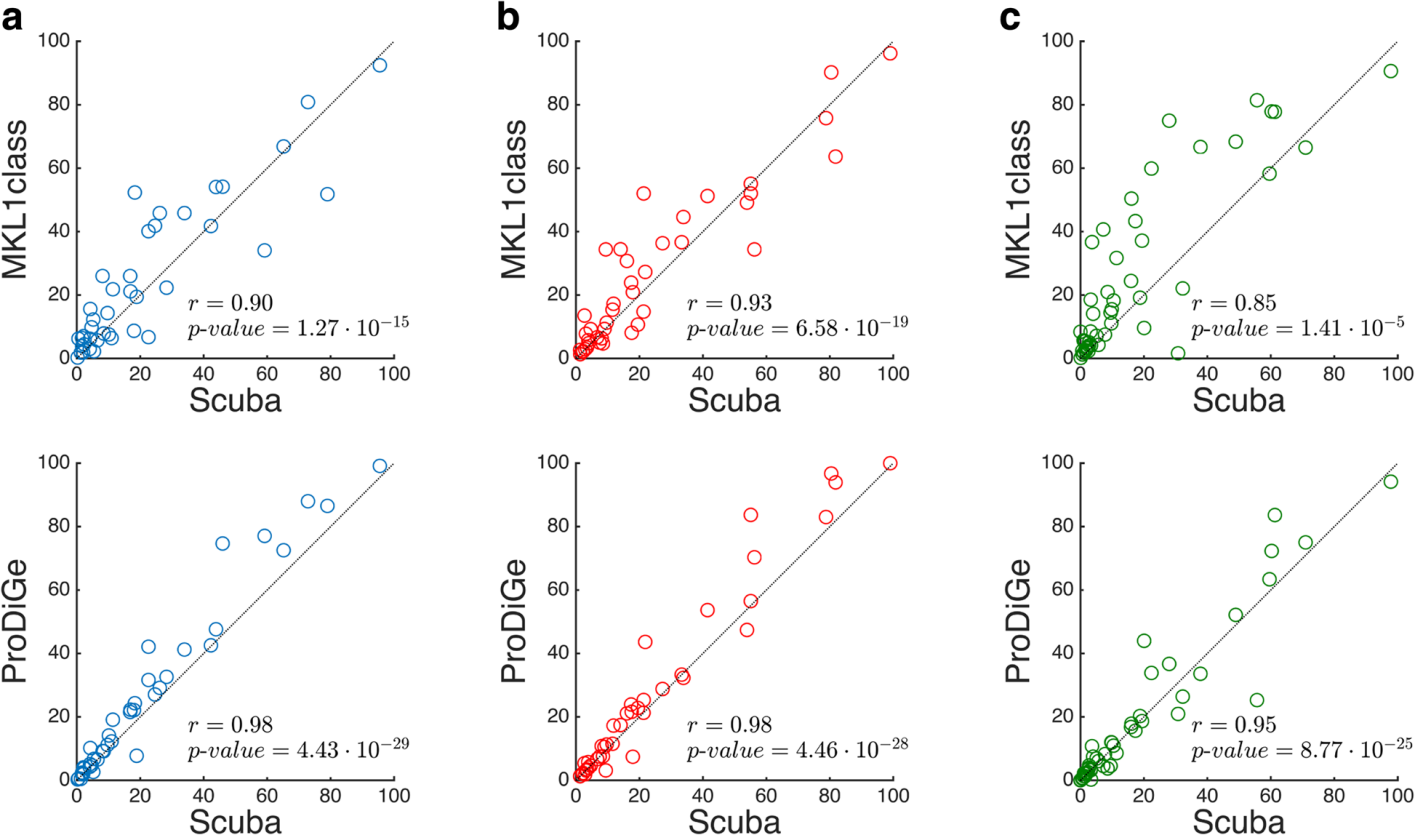
Comparison of normalized ranks predicted by Scuba and competing kernel methods. Normalized test genes rank distributions predicted by Scuba, MKL1class and ProDiGe for test genes in **(a)** genome-wide prioritizations in the unbiased evaluation of Table 2 - **(b)** candidate set-based prioritizations in the unbiased evaluation of Table 2 - **(c)** genome-wide prioritizations in the expanded unbiased evaluation of Table 4. In all cases, each point represents a test gene and lower values on the axes indicate better predictions. Genes lying on a diagonal have the same rank according to both methods considered on a plot. The further a gene lies above (below) a diagonal and the better it was ranked by Scuba (MKL1class/ProDiGe) compared to MKL1class/ProDiGe (Scuba). In each plot we show the Pearson correlation coefficient *r* between the test genes rank distributions and its associated *p*-value

Along with Scuba, we evaluated in this setting also MKL1class [12] and ProDiGe [13], two state-of-the-art kernel based gene prioritization methods. In Table 2 it is possible to see performances for all three methods. The significance of rank median differences between Scuba and competing methods was assessed by Wilcoxon signed rank tests, one for each comparison. At a significance threshold of 0.05, Scuba achieves significantly higher performances in genome-wide tasks compared to both baselines. In the candidate set-based setting, it performs significantly better than ProDiGe and better, although not significantly, than MKL1class. These differences can be visually appreciated in Fig. 1, where we compare the rank distributions of test genes obtained by the three methods. Scuba and MKL1class present moderate rank differences, particularly in the central region of the ranks. On the other hand, differences between Scuba and ProDiGe are smaller (Pearson *r*=0.98 in both cases) and almost all in favour of Scuba.

**Table 2:** Performances of Scuba, MKL1class and ProDiGe in the unbiased setting of Börnigen et al. [20]

Tool/Method	Rank	Rank	TPR in top	TPR in top	TPR in top	AUC	Rank difference
	median	average	5% (%)	10% (%)	30% (%)		*p*-value
Genome-wide prioritization methods							
Scuba	*10.55*	*20.48* ± 23.53	*33.3*	*47.6*	*78.6*	*0.80*	-
MKL1class [12]	13.30	23.42 ± 23.23	21.4	*47.6*	69.0	0.77	2.5 ·10^−2^ *
ProDiGe [13]	11.73	24.45 ± 27.33	31.0	45.2	71.4	0.76	3.0 ·10^−7^ *
Candidate set-based prioritization methods							
Scuba	*12.95*	*23.32* ± 25.46	*28.6*	*45.2*	*73.8*	*0.78*	-
MKL1class [12]	15.07	25.63 ± 24.73	23.8	40.5	61.9	0.76	9.7 ·10^−2^
ProDiGe [13]	14.41	26.39 ± 29.09	26.2	40.5	71.4	0.75	2.7 ·10^−3^ *

Values refer to predictions on all the 42 gene-disease associations. Rank difference *p*-values were obtained using Wilcoxon signed rank tests comparing separately Scuba/MKL1class and Scuba/ProDiGe ranks differences. Asterisks indicate significance of the tests at a threshold of 0.05

Italics indicates the top ranking score of each column

In Table 3 we show results for Scuba compared to the results obtained in the work of Börnigen et al., pertaining to eight prioritization systems [20]. In genome-wide predictions, Scuba dominates over the other tools. On predictions over smaller candidate sets, it is still competitive although best results are achieved by GeneDistiller [38], Endeavour [39] and ToppGene [40]. It is important to underline that in this case considered tools rely on different data sources, so we are comparing different prioritization systems rather different algorithms. Furthermore, tools are in some cases unable to provide an answer to a given task, depending on the underlying data sources (for more details see the original work [20]). We report the fraction of prioritizations on which tools are actually evaluated as response rate. This table has the purpose of showing the potentiality of Scuba relatively to what is easily accessible by non-bioinformaticians. However, since we used the String data for instance Scuba is directly comparable with Pinta [20, 41].

**Table 3:** Performances of Scuba and of some gene prioritization web tools in the unbiased setting of Börnigen et al. [20]

Tool/Method	Response	Rank	Rank	TPR in top	TPR in top	TPR in top	AUC
	rate (%)	median	average	5% (%)	10% (%)	30% (%)	
Genome-wide prioritization methods							
Scuba	100	*10.55*	*20.48* ± 23.53	*33.3*	*47.6*	*78.6*	*0.80*
Candid [44]	100	18.10	27.35 ± 24.62	21.4	33.3	64.3	0.73
Endeavour [39]	100	15.49	21.47 ± 22.37	28.6	38.1	71.4	0.79
Pinta [41]	100	19.03	23.52 ± 23.58	26.2	31.0	71.4	0.77
Candidate set-based prioritization methods							
Scuba	100	12.95	23.32 ± 25.46	28.6	45.2	73.8	0.78
Suspects [45]	88.9^a^	12.77^a^	24.64 ± 26.42^a^	33.3^a^	33.3^a^	63.0^a^	0.76^a^
ToppGene [40]	97.6	16.80	34.53 ± 35.31	*35.7*	42.9	52.4	0.66
GeneWanderer-RW [43]	88.1	22.10	29.55 ± 26.28	16.7	26.2	61.9	0.71
Posmed-KS [46]	47.6	31.44	42.07 ± 30.98	4.7	7.1	23.8	0.58
GeneDistiller [38]	97.6	*11.11*	*15.37* ± 13.77	26.2	*47.6*	78.6	*0.85*
Endeavour [39]	100	11.16	18.41 ± 21.39	26.2	42.9	*90.5*	0.82
Pinta [41]	100	18.87	25.23 ± 24.72	28.6	31.0	71.4	0.75

Response rate is the percentage of gene-disease associations considered by each tool. Values for Suspects were computed on the first 27 associations only (highlighted by ^a^)

Italics indicates the top ranking score of each column

**Table 4:** Performances of Scuba, MKL1class and ProDiGe in the expanded unbiased setting involving seven multifactorial diseases

Method	Rank	Rank	TPR in top	TPR in top	TPR in top	TPR in top	AUC	Rank difference
	median	average	1% (%)	5% (%)	10% (%)	30% (%)		*p*-value
Genome-wide prioritizations								
Scuba	8.13	*17.45* ± 22.33	*10.4*	41.7	*58.3*	*79.2*	*0.83*	-
MKL1class [12]	14.28	25.79 ± 26.96	2.1	27.1	45.8	66.7	0.74	1.2 ·10^−5^ *
ProDiGe [13]	*7.89*	18.40 ± 23.77	*10.4*	*43.8*	54.2	*79.2*	0.82	9.5 ·10^−2^

Values refer to predictions on 48 gene-disease associations. Rank difference *p*-values were obtained using Wilcoxon signed rank tests comparing separately Scuba/MKL1class and Scuba/ProDiGe ranks differences. Asterisks indicate significance of the tests at a threshold of 0.05

Italics indicates the top ranking score of each column

Next, we expanded this validation by employing gene-phenotype annotations derived from the Human Phenotype Ontology (HPO) [35]. This resource gathers information from several databases and makes available its monthly updates, permitting to trace the annotations history. We downloaded the HPO build 29 - dating March 2013 - and build 117 of February 2017. We compared the two annotations corresponding to these versions of HPO and extracted the gene-phenotype associations that were added in this time gap. We concentrated on phenotypes relative to the multifactorial diseases covered in the previous analysis, that could possibly have some previously undiscovered associations. We thus analyzed how the obtained genes are ranked in genome-wide prioritizations of the previous analysis, applying the same performance measures as before. The outcome is an analogous evaluation, but this time target genes are those extracted from HPO.

In Table 4 results for Scuba, MKL1class and ProDiGe are shown. We can observe a slightly different trend compared to previous results, with Scuba and ProDiGe having very close performance and MKL1class being significantly worse than Scuba. As a confirmation, in Fig. 1 we can see that there is no clear difference between test genes rank distributions for Scuba and ProDiGe. Instead, MKL1class ranks several test genes neatly lower compared to Scuba, with the associated Pearson correlation coefficient dropping to *r*=0.85.

## Discussion

Gene prioritization is progressively becoming essential in molecular biology studies. In fact, we are assisting to a continuous proliferation of a variety of *omic* data brought by technological advances. In the near future it is then likely that more heterogeneous knowledge will have to be combined. Moreover, the classes of biological agents to be prioritized are going to enlarge. For instance, we are only beginning to understand the complex regulation machinery involving non-coding RNA and epigenetic agents. It is estimated that around 90.000 human long non coding genes exist, whose functional implications are progressively emerging [42]. Facing these challenges, the development of novel methods is still strongly needed in order to enhance predictive power and efficiency.

Compared to the considered benchmark kernel methods - MKL1class and ProDiGe - Scuba has some important advantages. ProDiGe is one of the first proposed kernel-based PU learning method for gene prioritization [13]. It implements a PU learning strategy based on a biased SVM, which over-weights positive examples during training. In order to reach scalability to large datasets, it leverages a bagging procedure. Like ProDiGe, Scuba implements a learning strategy based on a binary classification set up, but from a different perspective. In a PU problem, the information on positive labels is assumed secure, while the information on negative labels is not. In terms of margin optimization, this translates in unbalanced entropy on the probability distributions associated to the two sets of training examples. It is then required to regularize more on the unlabelled class - having higher entropy - and in the limit of maximum uncertainty we get the uniform distribution.

MKL1class implements another effective approach for data integration, namely single class learning. This means that the model is obtained solely based on the distribution of known disease genes, disregarding unlabelled ones. Scuba has enhanced scalability compared to MKL1class, as it involves the optimization of the 1-norm of the margin vector from the different kernels. In contrast, MKL1class optimizes its 2-norm, which is more computationally demanding. Importantly, another distinctive feature of Scuba is a time complexity dependent on the number of positive examples and not on the number of total examples. As a consequence, Scuba can exploit the information on the whole data distribution and at the same time scale to large datasets without the need of sub-sampling the examples. This may be of great advantage as typically disease genes are orders of magnitude less numerous than the candidates.

Results from two different evaluation settings show that our proposed method Scuba outperforms many existing methods, particularly in genome-wide analyses. Compared to the two considered existing kernel-based methods, Scuba performances (considering AUC) are always higher, and often significantly higher. Moreover, Scuba has two main levels of scalability that make it particularly suitable for gene prioritization:

- **Scalability on number of kernels**: Scuba is able to deal with a large number of kernels defined on different data sources. As a consequence, it can be useful to get a more unified view of the problem and to build more powerful predicting models.
- **Scalability on number of training examples**: In typical gene prioritization problems, the number of known disease genes is much smaller than the number of candidates. Scuba is designed to efficiently deal with unbalanced settings and at the same time take advantage of the whole candidates distribution.

Altogether, our results show that Scuba is a valuable tool to achieve efficient prioritizations, especially in large-scale investigations. A detailed overview on the validation results for single diseases is available in Additional file 1: Tables S1, S3, S4.

Finally, as it is visible in Additional file 1: Table S2, performance with multiple kernels might be close to those with single kernels. Nevertheless, feeding multiple kernels into Scuba alleviates the issue of choosing appropriate kernels for each data source, as implemented in our work. Importantly, this strategy can also provide multiple views on the same data and possibly increase performance. Nevertheless caution must be paid since the more kernels are combined and the more parameters have to be learned, thus increasing the risk of over-fitting. We advice then to moderate the number of kernel matrices generated from each data source.

## Conclusion

In this work, we propose a novel computational kernel-based method to guide the identification of novel disease genes. Our method takes advantage of complementary biological knowledge by combining heterogeneous data sources. Every source can be transformed by appropriate kernel functions in order to take full advantage of its information. Our original algorithm is scalable relatively to the size of input data, number of kernel transformations employed and number of training examples. Experimental results support the thesis that Scuba is an effective approach and can be applied in various disease domains.

Scuba only requires a collection of input genes and optionally a set of candidate genes. The simple requirements make it applicable to a wide range of laboratory investigations. Furthermore, Scuba can be potentially employed also in other prioritization problems, as long as a PU approach and the integration of heterogeneous biological knowledge are needed.

## Additional file

Additional file 1 **Supplementary Tables.** PDF file containing experimental results for individual diseases and different kernel combinations. (PDF 158 kb)

## Abbreviations

AUC: Area under the receiver-operating-characteristic curve
DIR: Data integration rank
EasyMKL: Easy multiple kernel learning
F3PC: a logistic regression-based algorithm
HPO: Human phenotype ontology
HPRD: Human protein reference database
MDK: Markov diffusion kernel
MEDK: Markov exponential diffusion kernel
MKL: Multiple kernel learning
MRF: Markov random field
OMIM: Online Mendelian inheritance in man database
PCC: Pearson correlation coefficient
PU learning: Positive-unlabelled learning
RLK: Regularized Laplacian kernel
Scuba: Scalable Unbalanced gene prioritization
SVM: Support vector machine
TPR: True positive rate

## References

[1] Strachan T, Read A, Strachan T (2011). Human Molecular Genetics.

[2] Botstein D, Risch N (2003). Discovering genotypes underlying human phenotypes: past successes for mendelian disease, future approaches for complex disease. Nat Genet.

[3] Online Mendelian Inheritance in Man. http://omim.org/. Accessed 21 Dec 2016.

[4] Salgado D, Bellgard M, Desvignes J, Béroud C (2016). How to identify pathogenic mutations among all those variations: Variant annotation and filtration in the genome sequencing era. Hum Mutat.

[5] Moreau Y, Tranchevent L (2012). Computational tools for prioritizing candidate genes: boosting disease gene discovery. Nat Rev Genet.

[6] Ritchie M, Holzinger E, Li R, Pendergrass S, Kim D (2015). Methods of integrating data to uncover genotype-phenotype interactions. Nat Rev Genet.

[7] Shawe-Taylor J, Cristianini N (2004). Kernel Methods for Pattern Analysis.

[8] Gönen M, Alpaydın E (2011). Multiple kernel learning algorithms. J Mach Learn Res.

[9] Wang X, Xing E, Schaid D (2015). Kernel methods for large-scale genomic data analysis. Brief Bioinform.

[10] Borgwardt K, Ong C, Schönauer S, Vishwanathan S, Smola A, Kriegel H (2005). Protein function prediction via graph kernels. Bioinformatics.

[11] De Bie T, Tranchevent L, van Oeffelen L, Moreau Y (2007). Kernel-based data fusion for gene prioritization. Bioinformatics.

[12] Yu S, Falck T, Daemen A, Tranchevent L, Suykens J, De Moor B, Moreau Y (2010). L2-norm multiple kernel learning and its application to biomedical data fusion. BMC Bioinformatics.

[13] Mordelet F, Vert J (2011). Prodige: Prioritization of disease genes with multitask machine learning from positive and unlabeled examples. BMC Bioinformatics.

[14] Zakeri P, Elshal S, Moreau Y (2015). Gene prioritization through geometric-inspired kernel data fusion. 2015 IEEE International Conference on Bioinformatics and Biomedicine (BIBM).

[15] Chapelle O, Schölkopf B, Zien A (2006). Semi-supervised Learning.

[16] Aiolli F, Donini M (2015). Easymkl: a scalable multiple kernel learning algorithm. Neurocomputing.

[17] Aiolli F, Da San Martino G, Sperduti A (2008). A kernel method for the optimization of the margin distribution. International Conference on Artificial Neural Networks.

[18] Chen B, Li M, Wang J, Shang X, Wu F (2015). A fast and high performance multiple data integration algorithm for identifying human disease genes. BMC Med Genet.

[19] Devijver P, Kittler J (1982). Pattern Recognition: A Statistical Approach.

[20] Börnigen D, Tranchevent L, Bonachela-Capdevila F, Devriendt K, De Moor B, De Causmaecker P, Moreau Y (2012). An unbiased evaluation of gene prioritization tools. Bioinformatics.

[21] Kondor R, Lafferty J (2002). Diffusion kernels on graphs and other discrete structures. Proceedings of the 19th International Conference on Machine Learning.

[22] Chen B, Li M, Wang J, Wu F (2014). Disease gene identification by using graph kernels and markov random fields. Sci China Life Sci.

[23] Fouss F, Yen L, Pirotte A, Saerens M (2006). An experimental investigation of graph kernels on a collaborative recommendation task. Sixth International Conference on Data Mining.

[24] Chebotarev P, Shamis E (1997). The matrix-forest theorem and measuring relations in small social groups. Autom Remote Control.

[25] Polato M, Aiolli F (2016). Kernel based collaborative filtering for very large scale top-n item recommendation. Proceedings of the European Symposium on Artificial Neural Networks, Computational Intelligence and Machine Learning, ESANN.

[26] Chen Y, Wang W, Zhou Y, Shields R, Chanda SK, Elston RC, Li J (2011). In silico gene prioritization by integrating multiple data sources. PLoS ONE.

[27] Keshava Prasad TS, Goel R, Kandasamy K, Keerthikumar S, Kumar S, Mathivanan S, Telikicherla D, Raju R, Shafreen B, Venugopal A, Balakrishnan L, Marimuthu A, Banerjee S, Somanathan DS, Sebastian A, Rani S, Ray S, Harrys Kishore CJ, Kanth S, Ahmed M, Kashyap MK, Mohmood R, Ramachandra YL, Krishna V, Abdul Rahiman B, Mohan S, Ranganathan P, Ramabadran S, Chaerkady R, Pandey A (2009). Human protein reference database–2009 update. Nucleic Acids Res.

[28] Wu C, Orozco C, Boyer J, Leglise M, Goodale J, Batalov S, Hodge C, Haase J, Janes J, Huss J, Su A (2009). Biogps: an extensible and customizable portal for querying and organizing gene annotation resources. Genome Biol.

[29] Kanehisa M, Goto S (2000). Kegg: Kyoto encyclopedia of genes and genomes. Nucleic Acids Res.

[30] Vastrik I, D’Eustachio P, Schmidt E, Joshi-Tope G, Gopinath G, Croft D, de Bono B, Gillespie M, Jassal B, Lewis S, Matthews L, Wu G, Birney E, Stein L (2007). Reactome: a knowledge base of biologic pathways and processes. Genome Biol.

[31] Whirl-Carrillo M, McDonagh E, Hebert J, Gong L, Sangkuhl K, Thorn C, Altman R, Klein T (2012). Pharmacogenomics knowledge for personalized medicine. Clin Pharmacol Ther.

[32] Schaefer C, Anthony K, Krupa S, Buchoff J, Day M, Hannay T, Buetow K. Pid: the pathway interaction database. Nucleic Acids Res. 2008; 37(Database Issue):674–9. 10.1093/nar/gkn653 PMC2686461 18832364

[33] Jensen L, Kuhn M, Stark M, Chaffron S, Creevey C, Muller J, Doerks T, Julien P, Roth A, Simonovic M, Bork P, von Mering C (2009). String 8–a global view on proteins and their functional interactions in 630 organisms. Nucleic Acids Res.

[34] Goh K, Cusick M, Valle D, Childs B, Vidal M, Barabási A (2007). The human disease network. Proc Natl Acad Sci.

[35] Köhler S, Vasilevsky NA, Engelstad M, Foster E, McMurry J, Aymé S, Baynam G, Bello SM, Boerkoel CF, Boycott KM, Brudno M, Buske OJ, Chinnery PF, Cipriani V, Connell LE, Dawkins HJ, DeMare LE, Devereau AD, de Vries BB, Firth HV, Freson K, Greene D, Hamosh A, Helbig I, Hum C, Jähn JA, James R, Krause R, Laulederkind SJF, Lochmüller H, Lyon GJ, Ogishima S, Olry A, Ouwehand WH, Pontikos N, Rath A, Schaefer F, Scott RH, Segal M, Sergouniotis PI, Sever R, Smith CL, Straub V, Thompson R, Turner C, Turro E, Veltman MW, Vulliamy T, Yu J, von Ziegenweidt J, Zankl A, Züchner S, Zemojtel T, Jacobsen JO, Groza T, Mungall CJ, Haendel M, Robinson PN, Smedley D (2017). The human phenotype ontology in 2017. Proc Natl Acad Sci.

[36] Hanley J, McNeil B (1982). The meaning and the use of the area under a receiver operating characteristic (roc) curve. Radiology.

[37] Ensembl. http://www.ensembl.org/.

[38] Seelow D, Schwarz J, Schuelke M (2008). Genedistiller-distilling candidate genes from linkage intervals. PLoS ONE.

[39] Aerts S, Lambrechts D, Maity S, Van Loo P, Coessens B, De Smet F, Tranchevent L, De Moor B, Marynen P, Hassan B, Carmeliet P, Moreau Y (2006). Gene prioritization through genomic data fusion. Nat Biotech.

[40] Chen J, Xu H, Aronow B, Jegga A (2007). Improved human disease candidate gene prioritization using mouse phenotype. BMC Bioinformatics.

[41] Nitsch D, Gonçalves J, Ojeda F, de Moor B, Moreau Y (2010). Candidate gene prioritization by network analysis of differential expression using machine learning approaches. BMC Bioinformatics.

[42] Zhao Y, Li H, Fang S, Kang Y, Wu W, Hao Y, Li Z, Bu D, Sun N, Zhang M, Chen R (2016). Noncode 2016: an informative and valuable data source of long non-coding rnas. Nucleic Acids Res.

[43] Köhler S, Bauer S, Horn D, Robinson P (2008). Walking the interactome for prioritization of candidate disease genes. Am J Hum Genet.

[44] Hutz J, Kraja A, McLeod H, Province M (2008). Candid: a flexible method for prioritizing candidate genes for complex human traits. Genet Epidemiol.

[45] Adie E, Adams R, Evans K, Porteous D, Pickard B (2006). Suspects: enabling fast and effective prioritization of positional candidates. Bioinformatics.

[46] Yoshida Y, Makita Y, Heida N, Asano S, Matsushima A, Ishii M, Mochizuki Y, Masuya H, Wakana S, Kobayashi N, Toyoda T (2009). Posmed (positional medline): prioritizing genes with an artificial neural network comprising medical documents to accelerate positional cloning. Nucleic Acids Res.

