# Supplementary material for: Scuba: scalable kernel-based gene prioritization
Source: BMC Bioinformatics. 2018 Jan 25;19:23. doi: 10.1186/s12859-018-2025-5 (PMC5785908; doi:10.1186/s12859-018-2025-5)
Supplement: Additional file 1 — Supplementary Tables. PDF file containing experimental results for individual diseases and different kernel combinations. (PDF 158 kb) [file 12859_2018_2025_MOESM1_ESM.pdf]

Scuba: scalable kernel-based gene prioritization

Guido Zampieri, Dinh Van Tran, Michele Donini, Nicolò Navarin, Fabio Aiolli, Alessandro Sperduti, Giorgio Valle

Supplementary Material

Disease class	HPRD	BioGPS	Pathways	Average	Scuba
Cardiovascular	76.9	64.4	80.3	81.9	75.6
Connective	43.7	52.6	74.0	69.2	78.3
Dermatological	85.7	86.4	80.1	86.5	88.6
Developmental	67.3	54.1	65.3	66.7	79.7
Endocrine	71.7	69.5	72.9	78.6	78.4
Hematological	79.8	76.6	62.6	73.9	89.5
Immunological	89.8	75.8	96.3	96.4	96.4
Metabolic	79.6	72.8	90.4	90.7	96.1
Muscular	66.9	74.0	72.1	75.5	90.9
Ophthalmological	70.9	62.0	62.3	72.1	84.4
Renal	78.8	76.8	75.7	81.9	84.0
Skeletal	75.3	76.8	76.3	82.8	77.0
All	76.9	71.6	78.1	81.9	87.6

Table S1: Predictive performance in the setting of Chen *et al* [1] on 12 considered disease classes in terms of AUC and using different data sources.

Kernel function	kernel hyper-parameter	rank median	rank st.dev.	TPR at top 5% (%)	TPR at top 10% (%)	TPR at top 30% (%)	AUC
Genome-wide prioritizations							
$\mathbf{K}_{MD}$	2	11.13	24.36	31.0	47.6	73.8	0.78
	4	11.11	24.80	35.7	45.2	69.0	0.78
	6	12.34	25.16	33.3	45.2	73.8	0.78
	2,4,6	11.02	24.48	33.3	47.6	73.8	0.78
$\mathbf{K}_{RL}$	1	13.41	20.92	28.6	42.9	76.2	0.80
	10	11.77	21.15	31.0	42.9	78.6	0.81
	100	11.67	21.79	28.6	45.2	76.2	0.80
	1,10,100	12.40	20.90	28.6	40.5	76.2	0.80
Candidate set-based prioritizations							
$\mathbf{K}_{MD}$	2	13.20	26.70	23.8	45.2	69.0	0.77
	4	13.20	27.21	23.8	47.6	76.2	0.77
	6	14.19	27.31	23.8	42.9	73.8	0.77
	2,4,6	13.73	26.88	26.2	47.6	73.8	0.77
$\mathbf{K}_{RL}$	1	11.52	22.98	23.8	42.9	73.8	0.79
	10	11.45	23.25	26.2	47.6	76.2	0.80
	100	11.11	23.78	26.2	45.2	76.2	0.79
	1,10,100	11.60	23.15	23.8	40.5	73.8	0.79

Table S2: Scuba results in the experimental setting of Börnigen *et al* [2], using String v8.2 as data source and for different choices of kernels. From the third column to the last one: rank median and standard deviation, true positive rates (TPR) in the upper 5/10/30% of the ranking and average area-under-the-ROC-curve (AUC). Ranks are normalized in order to lie in the interval $]0, 100]$.

**Disease**	**Associated genes**	**genome-wide AUC**	**candidate set AUC**
Abdominal aortic aneurysm	ENSG00000136848	0.77	0.84
Alcohol dependence	ENSG00000148680	0.98	0.98
Arthrogryposis	ENSG00000152818	0.98	1.0
Asthma	ENSG00000182578	0.93	0.94
Autosomal recessive primary microcephaly	ENSG00000075702	0.41	0.44
Behcet's disease	ENSG00000136634	0.98	0.97
Bipolar schizoaffective disorder	ENSG00000146276<br>ENSG00000139618	0.97	0.98
Complex heart defect	ENSG00000121068	0.98	1.0
Congenital anomalies of the kidney and the urinary tract	ENSG00000164736<br>ENSG00000178188	0.97	0.96
Congenital diaphragmatic hernia	ENSG00000004961<br>ENSG00000154309	0.86	0.87
Crohn's disease	ENSG00000176920<br>ENSG00000185651<br>ENSG00000069399	0.89	0.89
Dursun syndrome	ENSG00000141349	0.58	0.46
Ehlers-Danlos syndrome	ENSG00000169105	0.99	1.0
Esophageal squamous cell carcinoma	ENSG00000138193<br>ENSG00000101276	0.3	0.23
Leprosy	ENSG00000111537	0.96	0.9
Lung adenocarcinoma	ENSG00000073282	0.89	0.84
Methylmalonic aciduria	ENSG00000167775	0.9	0.93
Metopic craniosynostosis	ENSG00000106571	0.98	0.98
Mitochondrial complex I deficiency	ENSG00000177646	0.95	0.96
Multiple sclerosis	ENSG00000120088	0.83	0.84
Myelodysplastic syndromes	ENSG00000106462	0.81	0.83
Nasopharyngeal carcinoma	ENSG00000085276<br>ENSG00000127863	0.81	0.8
Nonsyndromic cleft lip/palate	ENSG00000148175	0.82	0.8
Parkinson's disease	ENSG00000175104	0.82	0.8
Periventricular heterotopia	ENSG00000102103	0.54	0.45
Primary biliary cirrhosis	ENSG00000142606<br>ENSG00000142539	0.82	0.77
Psoriasis	ENSG00000056972	0.96	1.0
Retinal-renal ciliopathy	ENSG00000054282	1.0	1.0
Single-suture craniosynostosis	ENSG00000124813	0.98	0.98
Smooth pursuit eye movement abnormality	ENSG00000099901	0.27	0.2
Testicular germ cell tumor	ENSG00000137090<br>ENSG00000171681	0.5	0.41
Tetralogy of Fallot	ENSG00000145012	0.74	0.67
Type 2 diabetes	ENSG00000182247	0.21	0.19

Table S3: Scuba performance for single disorders considered by Börnigen *et al* in their evaluation of gene prioritization tools [2].

Disease	Associated genes	genome-wide AUC
Behcet's disease	ENSG00000162594<br>ENSG00000168811<br>ENSG00000138378<br>ENSG00000163823<br>ENSG00000136869<br>ENSG00000183542<br>ENSG00000164307<br>ENSG00000026103<br>ENSG00000206340<br>ENSG00000206450<br>ENSG00000134882	0.87
Bipolar schizoaffective disorder	ENSG00000175344<br>ENSG00000138592<br>ENSG00000151702<br>ENSG00000124782<br>ENSG00000171988<br>ENSG00000176986	0.68
Crohn's disease	ENSG00000140368	0.90
Parkinson's disease	ENSG00000064692<br>ENSG00000153234<br>ENSG00000116675<br>ENSG00000159082<br>ENSG00000184381<br>ENSG00000138246	0.89
Primary biliary cirrhosis	ENSG00000128604<br>ENSG00000181634<br>ENSG00000105329<br>ENSG00000110777<br>ENSG00000064419<br>ENSG00000016602<br>ENSG00000141076<br>ENSG00000106089<br>ENSG00000132912	0.76
Psoriasis	ENSG00000206237<br>ENSG00000196126<br>ENSG00000179344<br>ENSG00000206306<br>ENSG00000163599<br>ENSG00000206240<br>ENSG00000077150<br>ENSG00000141527<br>ENSG00000198246	0.94
Smooth pursuit eye movement abnormality	ENSG00000104133<br>ENSG00000171385<br>ENSG00000020922<br>ENSG00000070610<br>ENSG00000013503<br>ENSG00000167658	0.73

Table S4: Scuba performance for single disorders considered in Table 4 in the main text. These are the multifactorial diseases employed by Börnigen *et al* [2] with at least a new gene annotation between March 2013 and February 2017 as reported by the Human Phenotype Ontology [3].

Bibliography

[1] Chen, B and Li, M and Wang, J and Shang, X and Wu, FX, *A fast and high performance multiple data integration algorithm for identifying human disease genes*, BMC Medical Genomics, 8(3), S2 (2015)

[2] Börnigen, D and Tranchevent, LC and Bonachela-Capdevila, F and Devriendt, K and De Moor, B and De Causmaecker, P and Moreau, Y, *An unbiased evaluation of gene prioritization tools*, Bioinformatics, **28**(23), 3081-3088 (2012)

[3] Köhler, S and Vasilevsky, NA and Engelstad, M and Foster, E et al, *The Human Phenotype Ontology in 2017*, Nucleic Acids Research, **45**(D1), D865 (2017)
